# The role of Fluorine-18-Fluorodeoxyglucose positron emission tomography in staging and restaging of patients with osteosarcoma

**DOI:** 10.2478/raon-2013-0017

**Published:** 2013-05-21

**Authors:** Natale Quartuccio, Giorgio Treglia, Marco Salsano, Maria Vittoria Mattoli, Barbara Muoio, Arnoldo Piccardo, Egesta Lopci, Angelina Cistaro

**Affiliations:** 1 Department of Radiological Sciences, Nuclear Medicine Unit, University of Messina, Messina, Italy; 2 Department of Nuclear Medicine and PET/CT Centre, Oncology Institute of Southern Switzerland, Bellinzona, Switzerland; 3 Institute of Radiology, Catholic University of the Sacred Heart, Rome, Italy; 4 Institute of Nuclear Medicine, Catholic University of the Sacred Heart, Rome, Italy; 5 School of Medicine, Catholic University of the Sacred Heart, Rome, Italy; 6 Nuclear Medicine Unit, Galliera Hospital, Genoa, Italy; 7 Nuclear Medicine Unit, Humanitas Hospital, Milan, Italy; 8 Positron Emission Tomography Center IRMET, Euromedic inc., Turin, Italy. Coordinator of the AIMN PET-Pediatric Study Group

**Keywords:** osteosarcoma, bone sarcoma, ^18^F-Fluorodeoxyglucose, PET/CT, positron emission tomography

## Abstract

**Background:**

The objective of this study is to systematically review the role of positron emission tomography (PET) and PET/computed tomography (PET/CT) with Fluorine-18-Fluorodeoxyglucose (FDG) in patients with osteosarcoma (OS).

**Methods:**

A comprehensive literature search of published studies through October 10^th^, 2012 in PubMed/MEDLINE, Embase and Scopus databases regarding whole-body FDG-PET and FDG-PET/CT in patients with OS was performed.

**Results:**

We identified 13 studies including 289 patients with OS. With regard to the staging and restaging of OS, the diagnostic performance of FDG-PET and PET/CT seem to be high; FDG-PET and PET/CT seem to be superior to bone scintigraphy and conventional imaging methods in detecting bone metastases; conversely, spiral CT seems to be superior to FDG-PET in detecting pulmonary metastases from OS

**Conclusions:**

Metabolic imaging may provide additional information in the evaluation of OS patients. The combination of FDG-PET or FDG-PET/CT with conventional imaging methods seems to be a valuable tool in the staging and restaging of OS and may have a relevant impact on the treatment planning.

## Introduction

Osteosarcoma (OS) is the most common primary malignant bone tumour in children and adolescents, with a peak of incidence at the age of 15–19 years.^1^ OS is a tumour derived from primitive mesenchymal cells originating from bone and rarely from soft tissue.^2^ Although OS can occur in any bone, it is most common in the metaphyses of long bones: distal femur, proximal tibia, proximal humerus, and around the knee.^3^–^5^ OS has a high tendency to metastatic spread: 80% of all metastases arise in the lungs (20% of them at initial diagnosis) but metastases can also develop in bone and rarely in lymph nodes.^6^–^9^ The 5-year survival rate for OS patients with metastases is 20% compared to 65% for patients with localised disease.^10^

Usually, the treatment scheme for patients with OS is comprised of pre-operative chemotherapy, surgical removal of all detectable tumour sites and/or local treatment, followed by post-operative chemotherapy. The prognosis for patients with metastatic disease or recurrent disease remains poor.^11^,^12^ In order to correctly evaluate patients with OS in staging and restaging, a variety of diagnostic imaging modalities may be used, such as radiography, computed tomography (CT), magnetic resonance imaging (MRI), and bone scintigraphy.^13^ Fluorine-18-Fluorodeoxyglucose positron emission tomography (FDG-PET) has been successfully used to evaluate different malignant tumours^14^,^15^, such as musculoskeletal tumours.^16^ Tumour cells have a metabolic activity higher than normal cells and usually show an increased uptake of FDG, a glucose analogue. Like many other malignant tumours, OS have an increased rate of glycolysis, and consequently demonstrates an increased uptake of FDG. Standardised uptake value (SUV) can be used as semi-quantitative measure of the metabolic activity of a specific region of interest.^17^ Through the use of hybrid devices, integrating the high sensitivity of FDG-PET with the high spatial resolution of computed tomography (CT), a better diagnostic accuracy of PET/CT than PET and CT alone in detecting malignant tumours, such as OS, can be achieved.^16^–^18^ Several studies have shown the potential role of FDG-PET and PET/CT in the diagnosis of OS; however, a systematic review of published data in this field was lacking.

The purpose of this study is therefore to systematically review published data on the diagnostic performance of FDG-PET or PET/CT in patients with OS in order to assess the accuracy of these functional imaging methods in this setting.

## Materials and methods

### Search strategy

A comprehensive computer literature search of the PubMed/MEDLINE, Embase and Scopus databases was conducted in order to find relevant published articles on the diagnostic accuracy of FDG-PET and FDG-PET/CT in patients with osteosarcoma (OS). We used a search algorithm based on a combination of the terms: (a) “sarcoma” or “sarcomas “ or “osteosarcoma” or “osteogenic sarcoma “ or “bone sarcoma “ or “bone sarcomas” or “pediatric tumors” or “pediatric tumours” or “pediatric sarcomas” or “pediatric sarcoma” or “childhood sarcomas” or “bone tumors” or “bone tumours” or “osseous sarcomas” or “skeletal sarcomas” or “skeletal sarcoma” or “musculoskeletal sarcomas” and (b) “positron emission tomography” or “Positron emission Tomography And Computed Tomography” or “PET”. No beginning date limit was used; the search was updated until October 10^th^ 2012. Only articles in English language were selected. To expand our search, references of the retrieved articles were also screened for additional studies.

#### Study selection

Studies or subsets in studies investigating the diagnostic accuracy of FDG-PET or FDG-PET/CT in patients with OS were eligible for inclusion. Review articles, editorials or letters, comments, conference proceedings, articles not in the field of interest of this review, and case reports were excluded from this review. Only those studies or subsets in studies that satisfied all of the following criteria were included in the systematic review: (1) FDG-PET or FDG-PET/CT performed in patients with OS; (2) articles on the diagnostic accuracy of FDG-PET and FDG-PET/CT; (3) sample size of at least 10 patients with OS were included in the systematic review in order to select only the most relevant articles about the role of FDG-PET in osteosarcoma. Furthermore, this choice allowed reducing the publication bias. In fact, articles with a low number of patients usually report positive findings which further studies with a higher number of patients may exclude. When a possible overlap in patient data was found, the most complete article was included.

Four researchers (NQ, GT, MS and MVM) independently reviewed the titles and abstracts of the retrieved articles, applying the selection criteria mentioned above. Articles were rejected if they were clearly ineligible. The same four researchers then independently reviewed the full-text version of the remaining articles to determine their eligibility for the inclusion. Disagreements were resolved in a consensus meeting.

### Data extraction

For each included study in the systematic review, information was collected concerning the basic study (authors, year of publication, journal, country of origin), device used (PET or PET/CT), and patient characteristics (number of patients undergoing PET or PET/CT, mean age, sex, and number of patients with OS). Finally, the main findings of all articles included in this review are shown in the results.

## Results

### Literature search

The comprehensive computer literature search from the PubMed/MEDLINE, Embase, and Scopus databases revealed 28316 articles. Reviewing titles and abstracts, 13 articles comprising a total sample size of 289 patients with OS were selected applying the inclusion criteria mentioned above.^19^–^31^ These 13 studies were retrieved in their full-text version and included in this systematic review. No additional studies were found screening the references. The characteristics of the studies included are shown in Table 1.

## Literature data discussion

### Initial assessment: grading and staging

In 2000, Schulte *et al.*^19^ firstly demonstrated that FDG-PET, although never replacing biopsy, is a useful tool for estimating the biologic activity of skeletal lesions, including OS lesions. The authors evaluated the efficiency of FDG-PET in grading 202 patients with primary bone tumours, 44 of them with OS. FDG uptake was evaluated semi-quantitatively by determining the tumour-to-background ratio (T/B). Although sarcomas showed significantly higher T/B values than benign lesions, in a few cases it was not possible to discriminate between benign and malignant lesions. Using a T/B cut-off level of 3.0 for malignancy, the sensitivity (SS), specificity (SP), accuracy, positive predictive value (PPV), and negative predictive (NPV) of FDG-PET were 93%, 66.7%, 81.7%, 78.7% and 87.9%, respectively. No false negative findings occurred for patients with OS.^16^ In 2006, Kneisl and co-workers^20^ investigated the usefulness of FDG-PET in detecting occult non-pulmonary metastases at the initial work-up of 55 patients with bone sarcoma, 38 of them with OS. Only one of 38 OS patients (3%) has been upstaged by FDG-PET. Thus, according to the authors, in consideration of the high cost of the study, the ability of PET scan to detect occult non-pulmonary metastases has a minimal influence in the clinical management of OS patients at initial work-up.^20^ In 2009, Charest *et al*.^21^ evaluated the diagnostic performance of FDG-PET/CT for detection of soft tissue and osseous sarcomas in 212 patients, including 24 OS. SS of FDG-PET/CT for diagnosis of OS was 94.7%, detecting 12/12 tumours in the initial assessment and 6/7 tumours in the restaging, with mean SUV of 8.9.^21^ Further confirming results were documented in 2012 by Fuglø *et al.*^22^ who retrospectively studied a group of 89 patients with high-grade soft tissue sarcomas (59) and bone sarcomas (30, 14 of which were OS) in the initial assessment. Limiting the analysis to the detection efficiency of FDG-PET/CT for distant metastases from bone sarcoma the SS, SP, accuracy, PPV and NPV were 88%, 95%, 95%, 87% and 98%, respectively. In the lymph nodal based analysis FDG-PET/CT showed also high SS, SP, accuracy and NPV (100%, 90%, 91% and 100%, respectively) but a very low PPV (20%) due to confounding inflammatory tissue with high glucose metabolism in most of the patients of the study.^22^

### Comparison with conventional imaging: staging and restaging

In 1996, Garcia *et al.*^23^ compared the diagnostic accuracy of FDG-PET and ^99m^Tc-sestaMIBI scintigraphy in 48 patients with suspected recurrent/residual musculoskeletal sarcomas, including 18 OS. FDG-PET appeared to be more sensitive to ^99m^Tc-sestaMIBI scintigraphy in detecting active musculoskeletal sarcomas, with overall SS, SP, PPV and NPV of 98%, 90%, 98% and 90%, respectively.^23^

Völker *et al.*^24^ also evaluated the impact of FDG-PET for initial staging and therapy planning in 46 pediatric sarcoma patients, 11 of them with OS, demonstrating that the combination of FDG-PET with the conventional imaging is a valuable tool for the initial staging of OS and it has a relevant impact on therapy decisions. In fact, FDG-PET and conventional imaging reached the same efficiency in the detection of primary tumors (accuracy: 100%). In addition FDG-PET showed a higher SS than conventional imaging regarding the detection of nodal metastases (95% versus 25%) and bone metastases. In particular, bone scintigraphy showed a higher number of false-negative lesions compared with FDG-PET. Instead CT was more reliable than FDG-PET in depicting lung metastases, owing to their small size. Additionally combination of FDG-PET and conventional imaging changed the treatment planning in some cases.^24^ In the same year, Tateishi *et al.*^25^ demonstrated that the staging accuracy of combined PET/CT and conventional imaging is significantly higher than that of FDG-PET alone (p<0.0001). The authors retrospectively compared the diagnostic accuracy of FDG-PET/CT, FDG-PET and conventional imaging (bone scintigraphy, chest radiography, diagnostic CT of the chest and abdomen and locoregional MRI) in detecting nodal and distant metastases in a group of soft-tissue and bone sarcomas (including 19 OS). The standard of reference was histology or adequate follow-up. FDG-PET/CT showed to be superior to FDG-PET and conventional imaging in detecting nodal metastases. Similar results were documented comparing the ability of imaging modalities in detecting distant metastases. The authors conclude that the inclusion of FDG-PET/CT to the initial imaging work-up yields to a more accurate preoperative staging of bone and soft-tissue sarcomas (mainly because of the more accurate M-staging) and this is important in determining the appropriate treatment.^25^

In 2009, Piperkova *et al.*^26^ retrospectively reviewed 93 patients with bone and soft tissue sarcomas (15 of them with OS) who underwent FDG-PET/CT scan. The authors analyzed the results differentiating for FDG-PET alone, CT alone and combined FDG-PET/CT. For the initial staging, the combined FDG-PET/CT revealed the best performance, when compared with FDG-PET and CT alone, with a SS, SP, PPV, and NPV of 100%. Also for the re-staging group, the combined FDG-PET/CT revealed the best results with SS and SP of 100% and 95.9%, respectively. The authors concluded that in bone and soft tissue sarcomas for the initial staging and re-staging FDG-PET/CT has higher accuracy than FDG-PET and CT alone.^26^

In 2011, London *et al.*^27^ evaluated the performance of FDG-PET/CT compared to the conventional imaging in detecting malignant lesions with particular attention to lung metastases and predicting a histological response to chemotherapy in 41 children with primary bone tumours (20 patients with OS). On a lesion based analysis, the SS, SP, and accuracy of FDG-PET/CT were 81.8%, 97.5%, and 95.9%, respectively. In the lung lesion analysis, the SS, SP, and accuracy of FDG-PET/CT were 80.0%, 95.8%, and 93.0%, respectively. The authors concluded that FDG-PET/CT appears more accurate than the conventional imaging in detecting malignant lesions in childhood primary bone tumors, excluding lung lesions.^27^

In their prospective study in 2012, Bandopadhyaya *et al*.^28^ evaluated 22 biopsy proved OS patients undergoing FDG-PET/CT and ^99m^Tc-Dimercaptosuccinic acid (^99m^Tc-DMSA) whole body scintigraphy and compared the detection efficiency of the two imaging modalities. In detecting the primary lesion ^99m^Tc-DMSA scintigraphy showed the same SS (100%) of FDG-PET but lower SS in depicting lung metastases probably because the limited resolution of gamma camera respect to PET/CT, which instead reported a SS of 100%.^28^

### Comparison with conventional Imaging: evaluation of pulmonary lesions

Franzius *et al.*^29^ compared FDG-PET and spiral thoracic CT in detecting pulmonary metastases from malignant primary osseous tumors in 71 patients, including 32 patients with OS. In OS patients, FDG-PET revealed a SS, SP, and accuracy of 50%, 100%, and 92%, respectively. In all 71 patients (32 with OS and 39 with Ewing sarcoma), spiral thoracic CT revealed a SS, SP, and accuracy of 75%, 100%, and 94%, respectively. The authors concluded that spiral CT seemed to be superior compared to FDG-PET in detecting pulmonary metastases from malignant primary bone tumors, although a positive FDG-PET result can be used to confirm abnormalities seen on thoracic CT scans as neoplastic.^29^ In 2006, Iagaru *et al.*^30^ published a retrospective study of 106 patients with the histological diagnosis of osseous and soft tissue sarcomas (21 of them with OS), assessing the ability of FDG-PET and FDG-PET/CT versus chest CT in detecting pulmonary metastases. Overall, concordant PET and CT detection of pulmonary metastases was noted in 27 patients (67.5%). For all the patients, the SS and SP for FDG-PET were 68.3% and 98.4%, respectively. CT had a SS of 95.1% and SP of 92.3%. The authors demonstrated that CT of the chest was more sensitive than PET in detecting pulmonary metastases from OS; a significant portion of pulmonary nodules >1 cm on CT are PET-negative; sub-centimeter CT lesions should not be considered false positive if inactive on PET; a negative PET scan in the presence of suspicious CT findings in the chest cannot reliably exclude pulmonary metastases from osseous and soft tissue sarcomas.^30^ In 2012 Cistaro *et al*.^31^ studied 18 patients, 11 of which with OS, who had undergone FDG-PET/CT scan. They firstly attempted to find a SUVmax cut-off value helpful in discriminating the nature of the pulmonary nodules in pediatric bone sarcoma patients. They showed that a SUVmax threshold >1.09 was highly suggestive of malignancy when the nodule diameter was > 6 mm. No significant advantage was found in the semi-quantitative analysis (SUV max and SUV_ratio_) for the assessment of lesions below 6 mm. In the entire group of patients 18F-FDG-PET/CT had a SS of 90.3%, a SP of 87.5%, a PPV of 87.5%, and a NPV of 90.3% and an accuracy of 88.9%.^31^

## Conclusions and general remarks

From this systematic review on the role of FDG-PET and FDG-PET/CT in patients with OS, we are led to conclude that:

1. The combined metabolic and morphological information of FDG-PET/CT imaging allows a high diagnostic accuracy for the detection of OS; FDG-PET/CT is significantly more accurate than FDG-PET alone and improves staging and restaging in patients with OS.
2. With regard to the staging and restaging of OS, the SS, SP, and accuracy of FDG-PET and PET/CT seem to be high; FDG-PET and PET/CT seem to be superior to bone scintigraphy and conventional imaging methods in detecting bone metastases; conversely, spiral CT seems to be superior to FDG-PET in detecting pulmonary metastases from OS. A combination of FDG-PET/CT with conventional imaging methods is a valuable tool for staging and restaging of OS and may have a relevant impact on the treatment planning.
3. Most of the articles included in this systematic review evaluated the diagnostic accuracy of FDG-PET or PET/CT in mixed populations with different types of sarcomas, including some patients with OS. Further large prospective and multicenter studies evaluating the diagnostic accuracy of FDG-PET/CT in patients with OS are needed.

## Figures and Tables

**TABLE 1:** Characteristics of the studies included in the systematic review

**Authors**	**Year**	**Journal**	**Country**	**Study Design**	**Device used**	**Number of patients performing PET**	**Mean age (years)**	**Sex (%male)**	**Number of OS**
Garcia *et al.*^23^	1996	J Nucl Med	USA	Prospective	PET	48	40	50	18
Schulte *et al*.^19^	2000	J Nucl Med	Germany	Prospective	PET	202	28	63	44
Franzius *et al.*^29^	2001	Ann Oncol	Germany	Retrospective	PET	71	14	63	32
Iagaru *et al.*^30^	2006	Nucl Med Commun	USA	Retrospective	PET and PET/CT	106	45	49	21
Kneisl *et al*.^20^	2006	Clin Orthop Relat Res	USA	Retrospective	PET	55	NA	51	38
Tateishi *et al*.^25^	2007	Radiology	Japan	Retrospective	PET and PET/CT	117	42	59	19
Völker *et al*.^24^	2007	J Clin Oncol	Germany	Prospective	PET	46	13	52	11
Charest *et al.*^21^	2009	Eur J Nucl Med Mol Imaging	Canada	Retrospective	PET/CT	212	47	52	24
Piperkova *et al.*^26^	2009	Clin Nucl Med	USA	Retrospective	PET/CT	93	50	36	15
London *et al.*^27^	2011	Pediatr Radiol	Australia	Retrospective	PET/CT	41	13	63	20
Bandopadhyaya *et al*.^28^	2012	ISRN Oncol	India	Prospective	PET/CT	22	21	63	22
Cistaro *et al*.^31^	2012	Pediatr Blood Cancer	Italy	Retrospective	PET/CT	18	14	61	11
Fuglø *et al.*^21^	2012	Eur J Nucl Med Mol Imaging	Denmark	Retrospective	PET/CT	30	30	47	14

OS = osteosarcoma; NA = not available
